# Secondary metabolites of *Trichoderma* spp. as EGFR tyrosine kinase inhibitors: Evaluation of anticancer efficacy through computational approach

**DOI:** 10.1371/journal.pone.0296010

**Published:** 2024-01-24

**Authors:** H.G. Gowtham, Prasanna D. Revanasiddappa, Mahadevamurthy Murali, Sudarshana Brijesh Singh, M.R. Abhilash, Sushma Pradeep, Chandan Shivamallu, Raghu Ram Achar, Ekaterina Silina, Victor Stupin, Natalia Manturova, Ali A. Shati, Mohammad Y. Alfaifi, Serag Eldin I. Elbehairi, Shiva Prasad Kollur

**Affiliations:** 1 Department of PG Studies in Biotechnology, Nrupathunga University, Bangalore, Karnataka, India; 2 Department of Biotechnology, Siddaganga Institute of Technology, Tumkur, India; 3 Department of Studies in Botany, University of Mysore, Mysore, Karnataka, India; 4 Department of Studies in Environmental Science, University of Mysore, Mysore, India; 5 Department of Biotechnology and Bioinformatics, School of Life Sciences, JSS Academy of Higher Education & Research, Mysuru, Karnataka, India; 6 Division of Biochemistry, School of Life Sciences, JSS Academy of Higher Education and Research, Mysuru, Karnataka, India; 7 Department of Human Pathology, I.M. Sechenov First Moscow State Medical University (Sechenov University), Moscow, Russia; 8 Department of Hospital Surgery, NI. Pirogov Russian National Research Medical University, Moscow, Russia; 9 Biology Department, Faculty of Science, King Khalid University, Abha, Saudi Arabia; 10 School of Physical Sciences, Amrita Vishwa Vidyapeetham, Mysuru Campus, Mysuru, Karnataka, India; Ahram Canadian University, EGYPT

## Abstract

The present study explores the epidermal growth factor receptor (EGFR) tyrosine kinase inhibition efficacy of secondary metabolites in *Trichoderma* spp. through molecular docking, molecular dynamics (MD) simulation and MM-PBSA approach. The result of molecular docking confirmed that out of 200 metabolites screened, three metabolites such as Harzianelactone A, Pretrichodermamide G and Aspochalasin M, potentially bound with the active binding site of EGFR tyrosine kinase domain(PDB ID: 1M17) with a threshold docking score of ≤– 9.0 kcal/mol when compared with the standard EGFR inhibitor (Erlotinib). The MD simulation was run to investigate the potential for stable complex formation in EGFR tyrosine kinase domain-unbound/lead metabolite (Aspochalasin M)-bound/standard inhibitor (Erlotinib)-bound complex. The MD simulation analysis at 100 ns revealed that Aspochalasin M formed the stable complex with EGFR. Besides, the *in silico* predication of pharmacokinetic properties further confirmed that Aspochalasin M qualified the drug-likeness rules with no harmful side effects (*viz*., hERG toxicity, hepatotoxicity and skin sensitization), non-mutagenicity and favourable logBB value. Moreover, the BOILED-Egg model predicted that Aspochalasin M showed a higher gastrointestinal absorption with improved bioavailability when administered orally and removed from the central nervous system (CNS). The results of the computational studies concluded that Aspochalasin M possessed significant efficacy in binding EGFR’s active sites compared to the known standard inhibitor (Erlotinib). Therefore, Aspochalasin M can be used as a possible anticancer drug candidate and further *in vitro* and *in vivo* experimental validation of Aspochalasin M of *Trichoderma* spp. are required to determine its anticancer potential.

## Introduction

The epidermal growth factor receptor (EGFR) is a transmembrane protein located on some normal cells’ surfaces that regulates cell growth when the epidermal growth factor binds to it. It may also be found abnormally in high levels of certain human cancer cell types, which activates their growth and progression [1, 2]. Human EGFR inhibition could slow down or stop the growth of cancer cells [3]. The EGFR tyrosine kinase is crucial for the cellular signalling pathways regulating apoptosis, survival, growth, proliferation and differentiation [4]. The EGFR is considered one of the important protein targets in the development of anticancer drugs and therefore, the inhibition of EGFR is essential for the treatment of cancer to inhibit the progression and growth of EGFR-expressing tumor cells [2]. However, several EGFR inhibitors have been used to treat many types of cancers, such as pancreatic, lung, breast, thyroid, and colon cancer, caused by the up-regulation of EGFR [5]. Globally, the FDA approved EGFR tyrosine kinase inhibitors (*viz*., Erlotinib, Gefitinib, Almonertinib, Afatinib, Dacomitinib, Brigatinib, Icotinib, Lapatinib, Neratinib, Pyrotinib, Osimertinib, Olmutinib, Vandetanib and Simotinib)bind to EGFR tyrosine kinase domain and stop the EGFR activity [6, 7].

There are several reasons to conduct a study on the identification of EGFR inhibitors even when well-established inhibitors are available for clinical use (i) as over the time resistance to established inhibitors can develop; (ii) certain patient populations may not respond well to established inhibitors and (iii) they may experience severe side effects and identification of novel potential EGFR tyrosine kinase inhibitors is thus urgently required. Recent research efforts have been focused on developing new anticancer therapies that specifically target the EGFR signal transduction pathway, as the EGFR tyrosine kinase is involved in the initiation and progression of various cancers [8]. However, the mutation of EGFR has resulted rapidly in the development of clinical drug resistance to these EGFR inhibitors (especially Erlotinib and Gefitinib) [9, 10]. The available data can greatly help to design the new EGFR inhibitors from the diverse secondary metabolites associated with the plant and microbial sources. Apart from the plant derived compounds, the importance of secondary metabolites of microbes is reported in anticancer therapy using *in vitro* and *in silico* experiments [11, 12].

Microbial secondary metabolites such as antibiotics, anticancer agents, growth hormones, and pigments, etc., are the products with low molecular mass which are not required for microbial growth and possess excellent potential for improving animal and human health [13]. Among them, fungi and bacteria produce a wide variety of small bioactive molecules with important therapeutic properties. Studying the therapeutic potential of secondary metabolites produced by fungi has become the present research hotspot. *Trichoderma* spp. are fungi with the extraordinary ability to produce abundant secondary metabolites with great therapeutic potential [14]. It has been well documented that *in silico* evaluation of the secondary metabolites through molecular docking, molecular dynamic (MD) simulation and pharmacokinetic properties will have a substantial role in the detection of new metabolites of importance [15–17]. Based on the above facts, the main focus of the present study was to identify the new potential compounds from *Trichoderma* spp. as the inhibitors of EGFR tyrosine kinase through *in silico* molecular docking, MD simulation and MM-PBSA approach.

## Materials and methods

### Retrieval and preparation of secondary metabolites from *Trichoderma* spp.

The rationale for selecting 200 secondary metabolites produced by *Trichoderma* spp. as our focused target ligand molecules (S1 Table) is that they possess attractive chemical structures with significant biological activities such as antibacterial, antifungal, antiviral, herbicidal, nematicidal and insecticidal activity [14]. Erlotinib, a well-known EGFR inhibitor, was used as a standard inhibitor to compare the results better. The two-dimensional (2D) or three-dimensional (3D) structures of selected metabolites were downloaded from the PubChem database and ChemSpider database in Structure Data File (SDF) format. The MarvinSketch software was used to sketch the chemical structures of metabolites that were not found in any databases. The SDF format of structures was then converted into Protein Data Bank (PDB) format using Open Babel software [18]. The PRODRG server (https://davapc1.bioch.dundee.ac.uk/cgi-bin/prodrg/submit.html) optimized the geometry of the ligand PDB files prior to molecular docking. These optimized structures were then used for molecular docking studies as the ligand molecules.

### Retrieval and preparation of target protein

The crystal structure of EGFR tyrosine kinase domain with 4-Anilinoquinazoline inhibitor Erlotinib (PDB ID: 1M17) was obtained as a target protein from the Research Collaboratory for Structural Bioinformatics Protein Data Bank (RCSB PDB) website with a resolution of 2.60 Å [8, 19]. The 3D structure of the protein was prepared for performing the molecular docking using BIOVIA Discovery Studio Visualizer software (version 4.0) [20]. The ligand and water molecules found in the crystallized structure were deleted. After adding the protein’s missing hydrogen atoms and atomic solvation parameters, the Kollman united atom charges were assigned. The protein structure was subjected for energy minimization using Swiss-PDB Viewer software (version 4.1.0) to relieve steric clashes and optimize the structure’s geometry. After energy minimization, the more realistic and stable protein conformation was obtained for docking studies and the prepared protein molecule was further validated by Ramachandran plot analysis prior to molecular docking studies.

### Molecular docking studies

The molecular docking studies were performed using the AutoDock Vina program, which was implemented within the PyRx software (version 0.8) to predict the binding orientation and affinity of ligand molecules to a target protein [21]. A grid box was defined around the target protein to guide the docking calculations. The dimensions of the grid box were set to 93 × 66 × 51 Å in the x, y, and z directions, respectively. A grid spacing of 0.375 Å was used to define the search space for ligand binding. The exhaustiveness parameter was set to 100, which controls the thoroughness of the docking search with higher values indicating a more exhaustive search for potential binding poses. The binding energies resulting from the molecular docking were expressed in kilocalories per mole (kcal/mol). These energies provide an estimate of the binding strength between the ligand and the target protein. The lower binding energies typically indicate stronger binding. After the molecular docking, the results were visualized and analyzed using BIOVIA Discovery Studio Visualizer which allows examining and understanding the binding interactions formed between the protein and the ligand molecules including a standard drug used in the study. The obtained binding poses were further validated through self-docking or re-docking wherein the ligand is docked back into the protein’s binding site to confirm that the docking program can reproduce the known binding pose accuratelyin order to ensure the reliability of the docking results.

### Structural similarity calculation

DataWarrior software (version 5.5.0) with a default fingerprint descriptor FragFp was used to calculate the structural similarity between the compounds used in this study based on their canonical SMILES structure and binding behaviour.

### Pharmacokinetic studies

The pharmacokinetic studies were performed using the pkCSM-pharmacokinetic tool to calculate the absorption, distribution, metabolism, excretion and toxicity (ADMET) properties of lead molecules and their physicochemical properties during the process of drug discovery [22]. BOILED-Egg diagram and bioavailability radar map analysis were also achieved by using the SwissADME platform to assess the absorption and bioavailability of lead compound [23].

### Molecular dynamics simulation

After obtaining potential binding poses and affinity predictions from docking, the MD simulations are employed to understand the dynamic behaviour of the ligand-protein complex over time. The ligand-bound EGFR complex (with Aspochalasin M), the standard inhibitor-bound EGFR complex (with Erlotinib), and the unbound EGFR are all placed in a simulation box with appropriate solvent and ions. The GROMACS (Groningen Machine for Chemical Simulations) (Version 2018.1) biomolecular software program was used to execute the MD simulation [24, 25]. The SwissParam web-based tool was used to generate topologies and parameters for lead compound and standard inhibitor compatible with the CHARMM all atom force field. The pdb2gmx module of GROMACS was used to generate the topology files from a protein structure in PDB format based on the CHARMM36 force field (https://www.charmm.org/archive/charmm/resources/charmm-force-fields/) [26]. The cubic simulation box with a buffer distance of 1 Å was created to simulate the system in periodic boundary conditions. The TIP3P water model was used to mimic the behaviour of water in a real solution. The appropriate number of counter ions such as sodium ions (Na^+^) and chloride ions (Cl^–^) were also added to maintain a physiologically relevant environment.

After solvation, the system is likely to have high potential energy due to steric clashes and other factors. The steepest descent algorithm was commonly used to perform an energy minimization in order to relax the system and remove these high-energy configurations. The solvated system was equilibrated in two steps: NVT (canonical ensemble) and NPT (isothermal-isobaric ensemble). These steps ensure that the system reaches the desired temperature and pressure conditions before the actual production MD run. The 100 ps (picoseconds) indicates the length of time this equilibration simulation is run. The actual MD simulations were run for 100 ns at constant temperature (310 K) and 1 bar pressure [27]. In the simulation, the coordinates were saved for the entire system at regular intervals, in this case, every pico second. These saved coordinate snapshots, often referred to as trajectory frames, were used to analyze the system’s behaviour over time. The various analysis modules implemented in the GROMACS package were used for conducting conformational and structural analyses of MD simulation.

The relevant data were extracted from the MD trajectories which typically involves the RMSD (Root Mean Square Deviation), RMSF (Root Mean Square Fluctuation), Rg (Radius of Gyration), SASA (Solvent Accessible Surface Area), and hydrogen bond formation over time for each system (unbound EGFR, lead metabolite-bound EGFR, and standard inhibitor-bound EGFR). The data were saved in a format (e.g., CSV) that can be imported into QtGrace. The QtGrace’s GUI was used to create the plots for each analysis parameter. Finally, the generated plots were analyzed to draw conclusions about how the systems differ in terms of RMSD, RMSF, Rg, SASA, and hydrogen bond formation.

### MM-PBSA analysis

The g_mmpbsa package, which employs Molecular Mechanics Poisson−Boltzmann Surface Area (MM-PBSA) approach with GROMACS 2018.1 was employed to calculate the binding free energy of ligand-bound protein complex based on the result of MD simulation [28, 29]. The binding energy was calculated for the lead metabolite (Aspochalasin M) bound and known standard compound (Erlotinib) bound EGFR. The last 20 ns of the MD simulation trajectories were utilized to calculate the binding energy (ΔG_Binding_) with dt 1000 frames of the lead metabolite-bound protein complex by using the equations below:

$$\Delta G_{\text{Binding}}=G_{\text{complex}}-\left(G_{\text{Protein}}+G_{\text{Ligand}}\right)$$ (1)

$$\Delta \text{G}=\Delta {\text{E}}_{\text{MM}}+\Delta {\text{G}}_{\text{Solvation}}-\text{T}\Delta \text{S}=\Delta {\text{E}}_{\left(\text{Bonded}+\text{Non-bonded}\right)}+\Delta {\text{G}}_{\left(\text{Polart}+\text{Non-polar}\right)}-\text{T}\Delta \text{S}$$ (2)

where, G_Binding_ expresses the binding free energy, G_Complex_ expresses the total energy of lead metabolite/standard inhibitor bound target protein complex, and G_Protein_ and G_Ligand_ express the total energy of protein and ligand in water surrounded environment, respectively. ΔE_MM_expresses the average molecular mechanic’s potential energy in a vacuum, G_Solvation_ expresses the solvation energy, ΔE expresses the total energy of bonded plus non-bonded interactions, ΔG expresses the estimated binding free energy, ΔH expresses the estimated enthalpy contribution, ΔS expresses the change in system entropy upon ligand binding, T expresses the Temperature in Kelvin.

## Results and discussion

The use of small molecule inhibitors that specifically target the inhibition of the activity of human EGFR tyrosine kinase is considered a promising therapeutic approach for cancer treatment [3, 7]. The present study targeted EGFR tyrosine kinase suppression to identify the anticancer inhibitors from the secondary metabolites in *Trichoderma* spp. through *in silico* computational methods. The rationale for the selection of the crystal structure of EGFR tyrosine kinase domain with 4-Anilinoquinazoline inhibitor Erlotinib (PDB ID: 1M17) as our focused target protein is that the overexpression of EGFR has been associated with the advanced stages of numerous types of cancers, particularly lung, colon, breast, bladder and pancreatic cancers [8, 19].

### Molecular docking analysis

**Fig 1:**
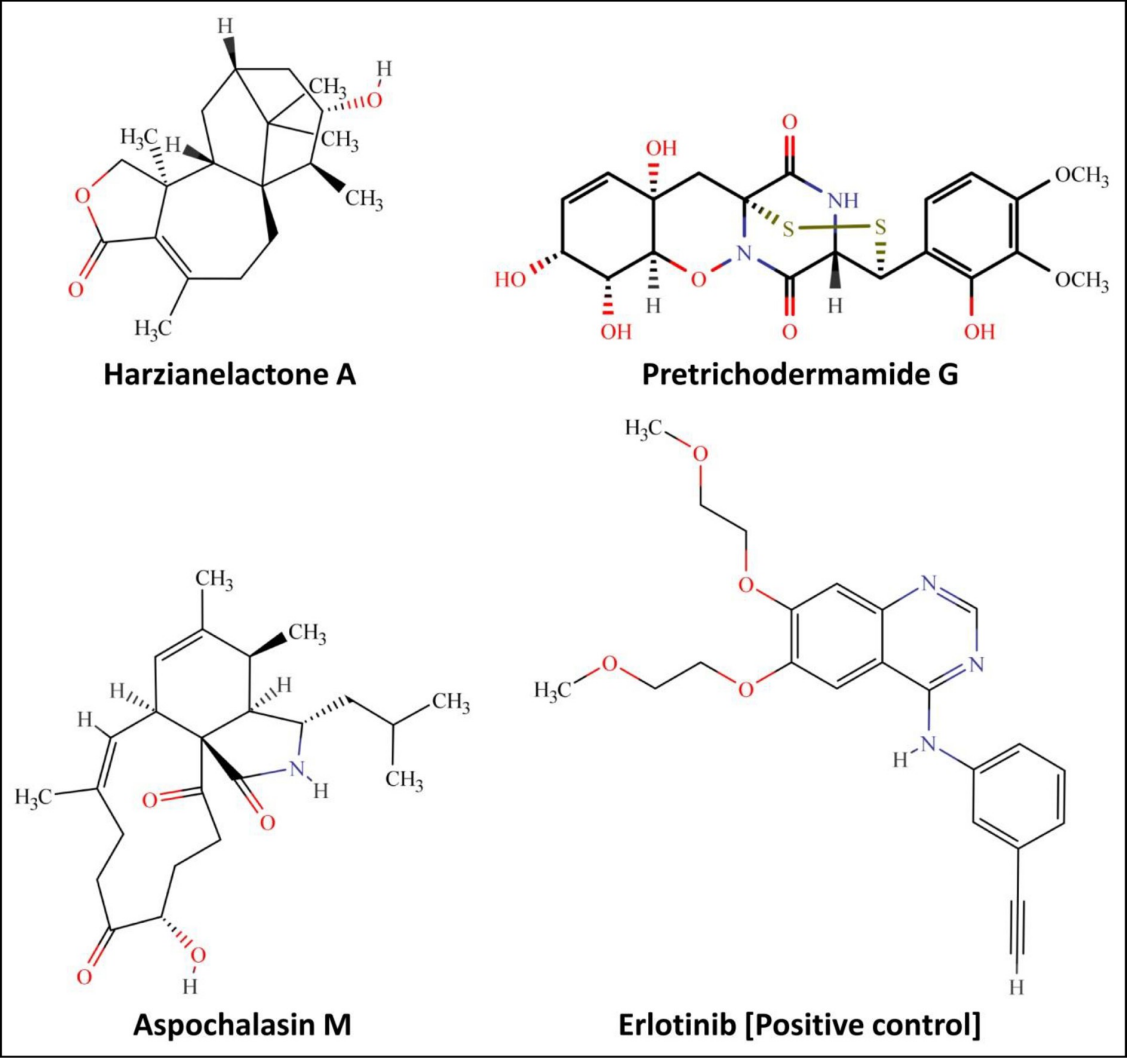
Structure of lead metabolites of *Trichoderma* spp. and standard inhibitor having the potential to bind at the active binding sites of EGFR tyrosine kinase domain.

**Fig 2:**
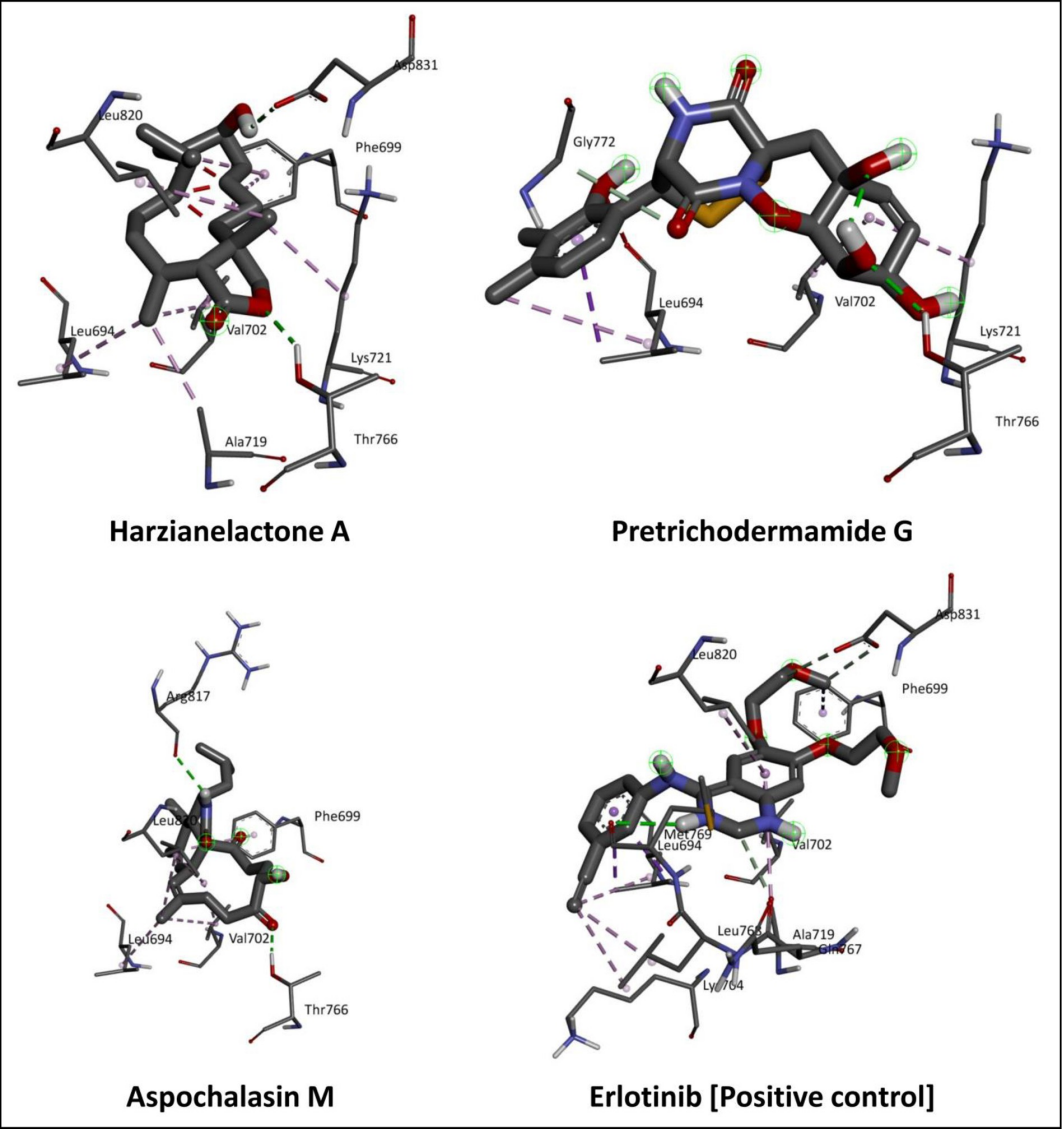
3D interaction of lead metabolites of *Trichoderma* spp. and standard inhibitor at the active binding sites of EGFR tyrosine kinase domain.

Docking studies are typically the first step in understanding how a ligand interacts with a target protein (EGFR). This involves predicting the preferred binding pose and affinity of the ligand within the protein’s binding site. Among the 200 secondary metabolites evaluated, three metabolites, namely Harzianelactone A (– 9.0 kcal/mol), Pretrichodermamide G (– 9.1 kcal/mol) and Aspochalasin M (– 9.4 kcal/mol) present in *Trichoderma* spp. showed the potential binding ability (cut-off value ≤– 9.0 kcal/mol) against EGFR compared to other metabolites and standard inhibitors. In the present study, the cut-off value ≤– 9.0 kcal/mol was considered the most effective threshold value of binding energy for validating correct posed molecules and selecting the top lead molecules. Docking scores of the metabolites against the targeted protein are tabulated in S1 Table. The standard EGFR inhibitor, Erlotinib, showed– 7.3 kcal/mol binding energy. The 2D structures of lead and standard compounds against EGFR tyrosine kinase domain are represented in Fig 1. The binding pose of top lead EGFR tyrosine kinase inhibitors and the standard compound is depicted in Fig 2. The molecular docking studies provide valuable insights into the amino acids that are crucial for ligand inhibition which is crucial for rational drug design and understanding the molecular basis of biological processes. The identification of amino acids that are important for the inhibition based on molecular docking results typically involves analyzing the binding interactions between a ligand (e.g., a drug or small molecule) and a target protein (e.g., an enzyme or receptor) [30].

**Fig 3:**
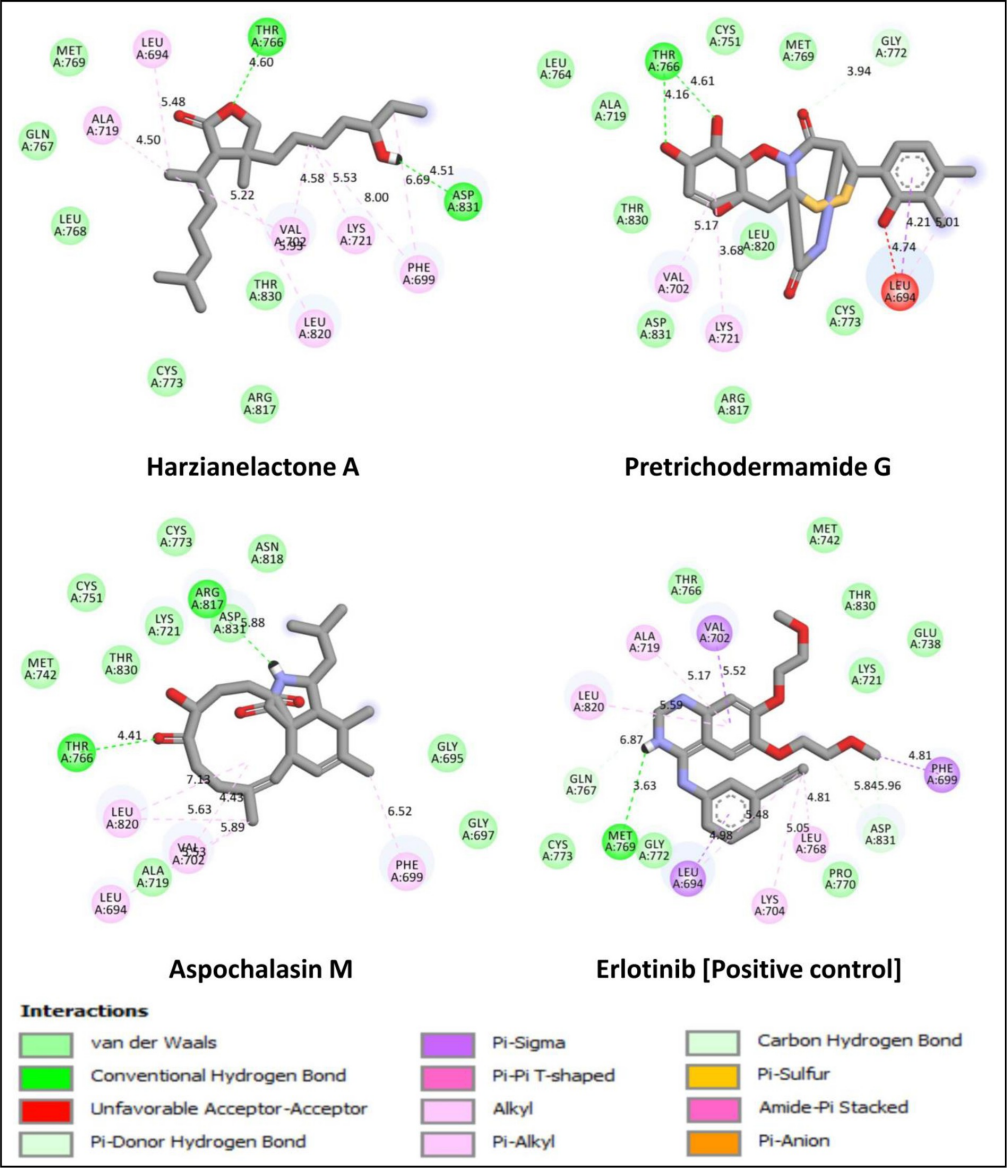
2D interaction of lead metabolites of *Trichoderma* spp. and standard inhibitor at the active binding sites of EGFR tyrosine kinase domain.

During the study, Aspochalasin M formed hydrogen bonds with THR766 and ARG817 amino acid residues of the EGFR tyrosine kinase domain (Fig 3). The hydrogen bond formation with these amino acids plays a vital role in stabilizing the protein ligand binding and overall binding affinity and specificity thereby leading to enzyme inhibition. When a ligand forms hydrogen bonds with specific amino acid residues, it helps to lock the ligand into the binding pocket and enhances the strength of the interaction [31]. The results indicate that amino acid THR766 has polar or hydrogen bond donor/ acceptor groups within its side chain and when the ligand (Aspochalasin M) forms a hydrogen bond with THR766, it leads to the stabilization of the enzyme EGFR tyrosine kinase domain. Likewise, ARG817, an arginine residue, has a positively charged guanidine group that helps participate in hydrogen bond interactions through their amino groups with electronegative groups on the ligand (such as oxygen atoms), thereby enhancing the binding affinity. From the studies of Stamos et al. [19], it may be observed that the H-bond formation with native ligand (Erlotinib) in the EGFR protein structure was observed with MET769 and THR766. In addition, they have also noted that a water molecule was required in order to bridge the H-bond for THR766. But during the present study, as the H-bond was formed readily with the THR766 with all the three potential inhibitors and hence additional water molecule may not be required/ play any role in bridging the hydrogen bonds with THR766. Moreover, Aspochalasin M showed hydrophobic interactions at the active site of targeted EGFR during the study and these hydrophobic interactions are known to play a crucial role in enhancing binding specificity [32]. The results highlight the potential of specific metabolite, especially Aspochalasin M, as a strong EGFR inhibitor based on its binding energies and interaction patterns.

In addition, during the re-docking, the RMSD values of the selected compounds were ≤ 2.0 Å in the present study, which agree with the findings of Ramirez and Caballero [33], wherein ≤ 2.0 Å of RMSD corresponds to the good docking solutions. In the virtual screening of 329 naturally occurring plant-based flavonoids, six flavonoids were found to be potential EGFR inhibitors with good docking scores [34]. Sepay et al. [35] have reported that Rhamnocitrin derivative Tupichinols E from *Tupistra chinensis* showed 1.4 times more binding affinity with EGFR tyrosine kinasethan Osimertinib (a well-known EGFR inhibitor). The four quinoxalinone containing compounds such as CPD4, CPD15, CPD16 and CPD21 were promising to possess a lower than −7.0 kcal/mol compared to the reference drug (Osimertinib) towards the tyrosine kinase domain of EGFR [36].

### Structural similarity calculation

**Fig 4:**
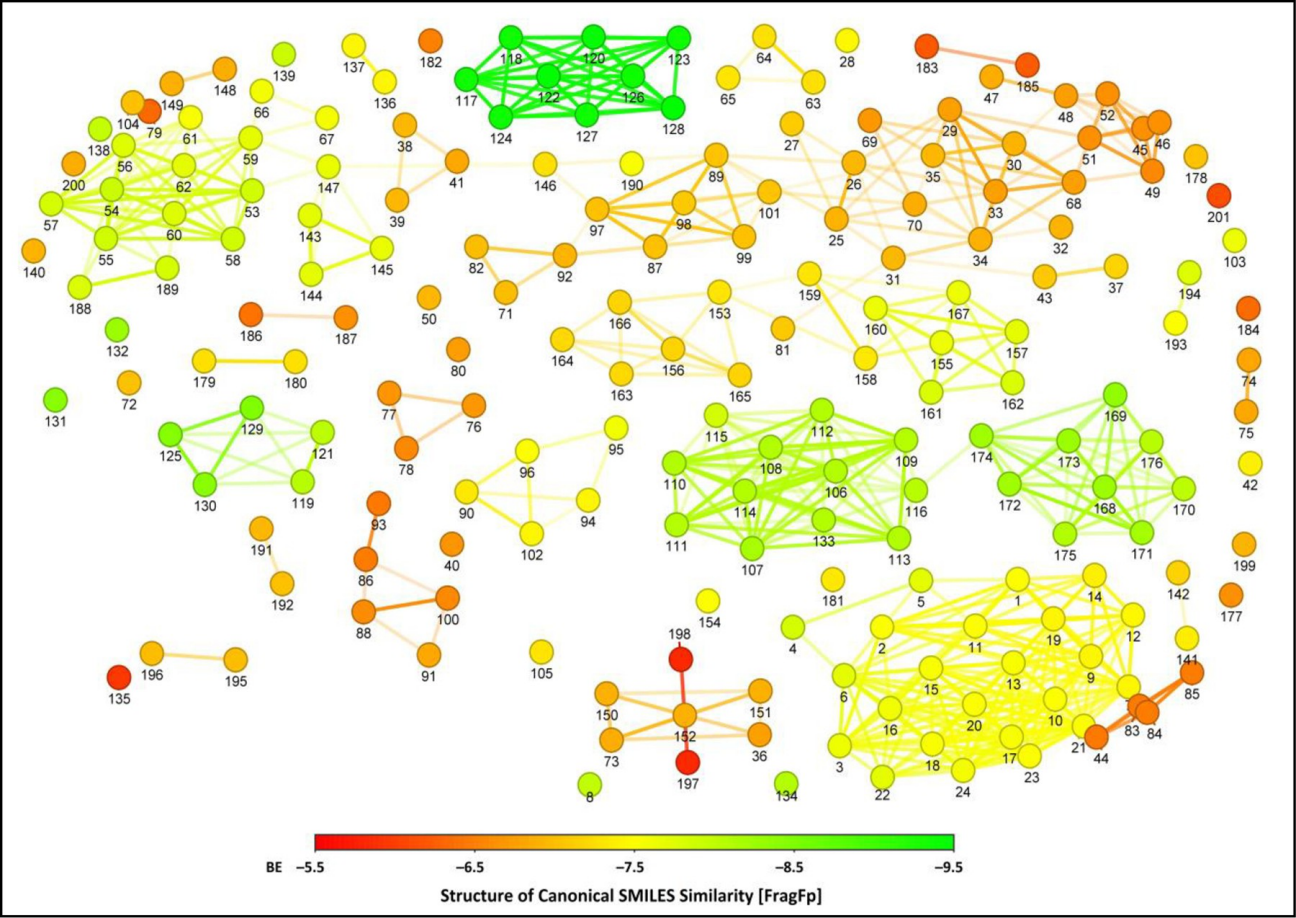
Structural similarity of 200 compounds and standard drug used in the study. Lines between the dots represent the structurally similar compounds and the numbers on each dot represent the compound name, as listed in S1 Table.

The structurally similar ligand molecules will bind to the same/identical biological targets and occupy the same region in the binding sites of protein receptors, thereby supporting in designing strategies by shape similarity for their potential application during the drug discovery process [37, 38]. This matching between the structurally similar ligand molecules may indicate the similar pharmacological action at the protein receptor. All the selected compounds and Erlotinib were further used to calculate structural similarity based on the correlation between the compound’s canonical SMILES structure similarity and binding behaviour. Fig 4 represents the more relevant range above 80% structural similarity. The compounds were arranged by their structural similarity relationship and similar binding affinity range. Thestudy’s structurally similar ligands offered similar binding affinity to the target protein (EGFR tyrosine kinase) as they occupied the same 3D position in the binding sites of the protein receptor, as noticed in the studies of Gowtham et al. [29].

### Pharmacokinetic studies

The physicochemical and pharmacokinetics properties of lead metabolites of *Trichoderma* spp. and standard EGFR inhibitor (Erlotinib) are predicted during the drug development process. Lipinski’s rule of five is the most popular method for determining the drug-likeness properties, which predict the ability of compounds to be active orally in the human body. This rule helps to evaluate the drug-likeness properties *viz*., molecular weight (expressed as g/mol), H-bond donors-acceptors, rotatable bonds and log P value. According to the rule, any drug-like compound must have a molecular weight ≤ 500 g/mol, 5 H-bond donors, 10 H-bond acceptors, 10 rotatable bonds and a calculated partition coefficient (log P) >5. The results of physicochemical properties are presented in Table 1.

**Table 1:** Physicochemical properties of lead metabolites of *Trichoderma* spp. and standard inhibitor.

Descriptor	Harzianelactone A	Pretrichodermamide G	Aspochalasin M	Erlotinib
Molecular weight (g/mol)	318.457	466.537	401.547	393.443
log P	3.7092	0.19504	3.3651	3.4051
Rotatable bonds	0	1	2	10
Hydrogen bond acceptors	3	9	4	7
Hydrogen bond donors	1	5	2	1
Polar surface area (Å^2^)	139.03	185.351	173.565	169.532

The prediction of ADMET properties is an essential criterion during the drug discovery process since they are responsible for around 60% of clinical drug development failure due to their poor drug-like properties [39]. Initially, the ADMET properties are predicted using *in silico* tools in the drug development to filter out the compounds from the pipeline with poor ADMET properties, hence decreasing the cost of research and development. In addition, the ADME studies offered scientific evidence that the potential metabolites are clinically prevalent, thereby supporting the use of the same metabolites for further *in vitro* and *in vivo* studies (Table 2). Regarding the absorption properties, Aspochalasin M was found to have a promising oral availability attributable to its optimal Caco-2 cell permeability (>0.9), intestinal absorption (>90%) and skin permeability (log Kp <−2.5 cm/h). The volume of distribution (VDss) and fraction unbound (Fu) are the most imperative pharmacokinetic properties of drugs [40]. The VDss values determine the extent of drug distribution, while the unbound fraction determines the amount of unbound drug in plasma that is free to discharge. A VDss value of >0.45 predicts drug distribution in tissue, but a VDss value of <−0.15 predicts drug distribution in plasma. Aspochalasin M, which shows the intermediate VDss value range (– 0.016), had an adequate distribution in plasma with an unbound fraction (0.084). These results showed that Aspochalasin M had a good plasma distribution and significant unbound fraction, allowing it to interact with the drug’s pharmacological target.

**Table 2:** Predicted pharmacokinetic and toxicity properties of lead metabolites of *Trichoderma* spp. and standard inhibitor.

	Model Name	Harzianelactone A	Pretrichodermamide G	Aspochalasin M	Erlotinib
**Absorption**	Water solubility (log mol/L)	– 4.71	– 3.213	– 4.353	– 4.403
	Caco-2 permeability (log P_app_ in 10^−6^ cm/s)	1.374	0.583	1.313	1.238
	Human intestinal absorption (% Absorbed)	95.818	57.987	96.811	95.549
	Skin permeability (log Kp in cm/h)	– 3.569	– 2.775	– 3.341	– 2.738
	P-glycoprotein substrate	No	Yes	Yes	No
	P-glycoprotein I inhibitor	Yes	No	Yes	Yes
	P-glycoprotein II inhibitor	No	No	Yes	Yes
**Distribution**	Human VDss (log L/kg)	0.408	0.263	– 0.016	– 0.053
	Human fraction unbound (Fu)	0.179	0.326	0.084	0.04
	BBB permeability (log BB)	0.283	– 1.469	0.3	– 0.67
	CNS permeability (log PS)	– 2.573	– 3.99	– 1.879	– 3.384
**Metabolism**	CYP2D6 substrate	No	No	No	No
	CYP3A4 substrate	Yes	No	Yes	Yes
	CYP1A2 inhibitor	No	No	No	Yes
	CYP2C19 inhibitor	No	No	No	Yes
	CYP2C9 inhibitor	No	No	No	Yes
	CYP2D6 inhibitor	No	No	No	No
	CYP3A4 inhibitor	No	No	No	Yes
**Excretion**	Total clearance (log mL/min/kg)	0.596	0.041	0.804	0.591
	Renal OCT2 substrate	Yes	No	Yes	No
**Toxicity**	AMES toxicity	No	No	No	No
	Human Max. tolerated dose (log mg/kg/day)	– 0.398	– 0.428	– 0.723	0.002
	hERG I inhibitor	No	No	No	No
	hERG II inhibitor	No	No	No	Yes
	Oral Rat Acute Toxicity (LD_50_) (mol/kg)	1.82	4.024	2.638	2.368
	Oral Rat Chronic Toxicity (LOAEL) (log mg/kg bw/day)	1.728	3.227	1.76	0.88
	Hepatotoxicity	No	Yes	No	Yes
	Skin sensitisation	No	No	No	No
	*Tetrahymena pyriformis* toxicity (log μg/L)	0.914	0.285	0.449	0.334
	Minnow toxicity (log mM)	0.662	3.197	0.751	– 0.437

In addition, Aspochalasin M with log PS value = > –3 suggested that it could penetrate the central nervous system (CNS) compared to the standard inhibitor (Erlotinib). The predicted total clearance values determine the capacity of the body to remove the drug. It was indicated that Aspochalasin M has a good renal elimination (0.804 mL/min/kg) and was the renal organic cation transporter 2 (OCT2) substrate compared to Erlotinib. To explore the adverse detrimental effects of compounds on humans, it is necessary to assess their toxicology. Therefore, it is another crucial step in the drug discovery process. In our study, toxicity tests revealed that Aspochalasin M showed non-mutagenicity with no harmful side effects (*viz*., hERG toxicity, hepatotoxicity and skin irritation) and exhibited favourable logBB value. It was also discovered that the acute toxicity level (LD_50_) of Aspochalasin M to cause death was 2.638 mol/kg. Finally, Aspochalasin M was found to pass the *T*. *pyriformis* and Minnow toxicity tests compared to the standard inhibitor (Erlotinib).

**Fig 5:**
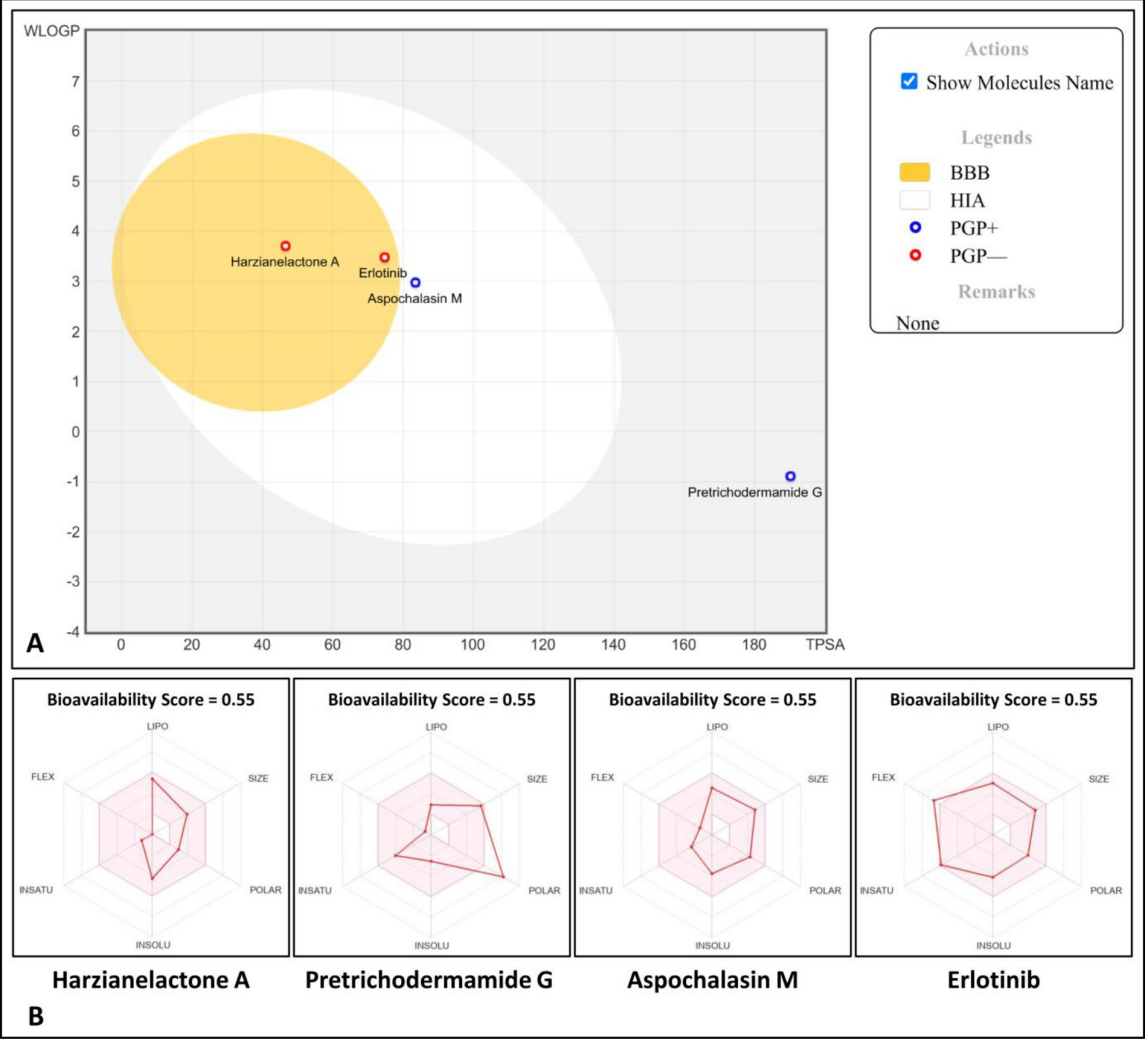
BOILED-Egg diagram (A) and bioavailability radar map (B) of lead metabolites of *Trichoderma* spp. and standard inhibitor. The pink region within the hexagon shows the optimal range of each drug-likeness property. Lipophilicity (LIPO) as XLOGP3 is ranged between −0.7 and +5.0, molecular weight (SIZE) is ranged between 150 and 500 g/mol and polar surface area (POLAR) as topological polar surface area (TPSA) is ranged between 20 and 130 Å^2^), insolubility (INSOLU) in water by log S scale is not > 6, in saturation (INSATU) as the fraction of carbons in sp^3^ hybridization is ranged between 0.25 and 1, and flexibility (FLEX) of rotatable bonds is not > 9 [23].

The important factors in improving bioactivity and overall body health are the absorption and metabolism of the drug in the human body and the drug’s bioavailability to target cells. The pharmacokinetic properties affect the drug permeability across the various physiological barriers (*viz*., membrane permeability, blood–brain barrier penetration and gastrointestinal absorption) in humans. The BOILED-Egg model quickly predicts passive blood–brain barrier penetration and gastrointestinal absorption of bioactive molecules in the drug-designing process. The BOILED-Egg diagram prepared for the lead metabolites (*viz*., Harzianelactone A, Pretrichodermamide G and Aspochalasin M) present in *Trichoderma* spp. and standard inhibitor (Erlotinib) showed satisfactory results (Fig 5A). The chemicals that are anticipated to passively cross the blood–brain barrier are located in the yolk (yellow) area of BOILED-Egg. It was assumed that Harzianelactone A and Erlotinib would passively cross the blood–brain barrier because they are found in the yolk of BOILED-Egg. The chemicals that are anticipated to be absorbed passively by the gastrointestinal tract are located in the white (albumin) region of BOILED-Egg. It was hypothesized that Aspochalasin M would have superior absorption in the digestive system because it is found in the white of BOILED-Egg. The blue dots represent the chemicals that P-glycoprotein is expected to remove from the CNS. The P-glycoprotein was anticipated to remove Aspochalasin M and Pretrichodermamide G from the CNS. The red dots represent the chemicals that P-glycoprotein is expected not to remove from the CNS. It was predicted that P-glycoprotein wouldn’t remove Harzianelactone A and Erlotinib from the CNS.

Protein-drug binding that influences the bioavailability and distribution of active compounds is a major limiting factor in the absorption of drugs across biological barriers [41]. Besides, the oral bioavailability of the drug is also a crucial and critical factor in drug design. A higher score lowers the amount of drug that must be delivered to achieve its anticipated pharmacological effect and vice-versa, thereby lowering the risk of side effects and toxicity [16, 17]. The higher absorption of the molecules’ gastrointestinal tract naturally leads to improved bioavailability. Based on the observed results, the top three binding scored metabolites (such as Harzianelactone A, Pretrichodermamide G and Aspochalasin M) and standard inhibitor (Erlotinib) had a considerable gastrointestinal absorption with a positive bioavailability score of 0.55 (Fig 5B). As a result, Aspochalasin M may improve its absorption in the digestive system when administered orally. The results highlighted that the lead metabolite Aspochalasin M could be an excellent possible drug-like candidate for cancer treatment and could lead to further studies.

### Molecular dynamics simulation

Based on docking results, the MD simulation was run on an unbound EGFR tyrosine kinase domain, Aspochalasin M-bound EGFR tyrosine kinase domain system, to study the dynamic behaviour of targeted protein in a solvated environment with respect to time. Simultaneously, the experimentally validated inhibitor (Erlotinib) of EGFR was also employed to compare the results. In this investigation, three simulations with unbound protein and representative compounds bound protein complex were run at 100 ns time. The simulation results presented the analysis of RMSD, RMSF, Rg and SASA of protein-ligand complex, the number of ligand hydrogen bonds maintained during MD simulation and variations in protein secondary structure and their complexes.

**Fig 6:**
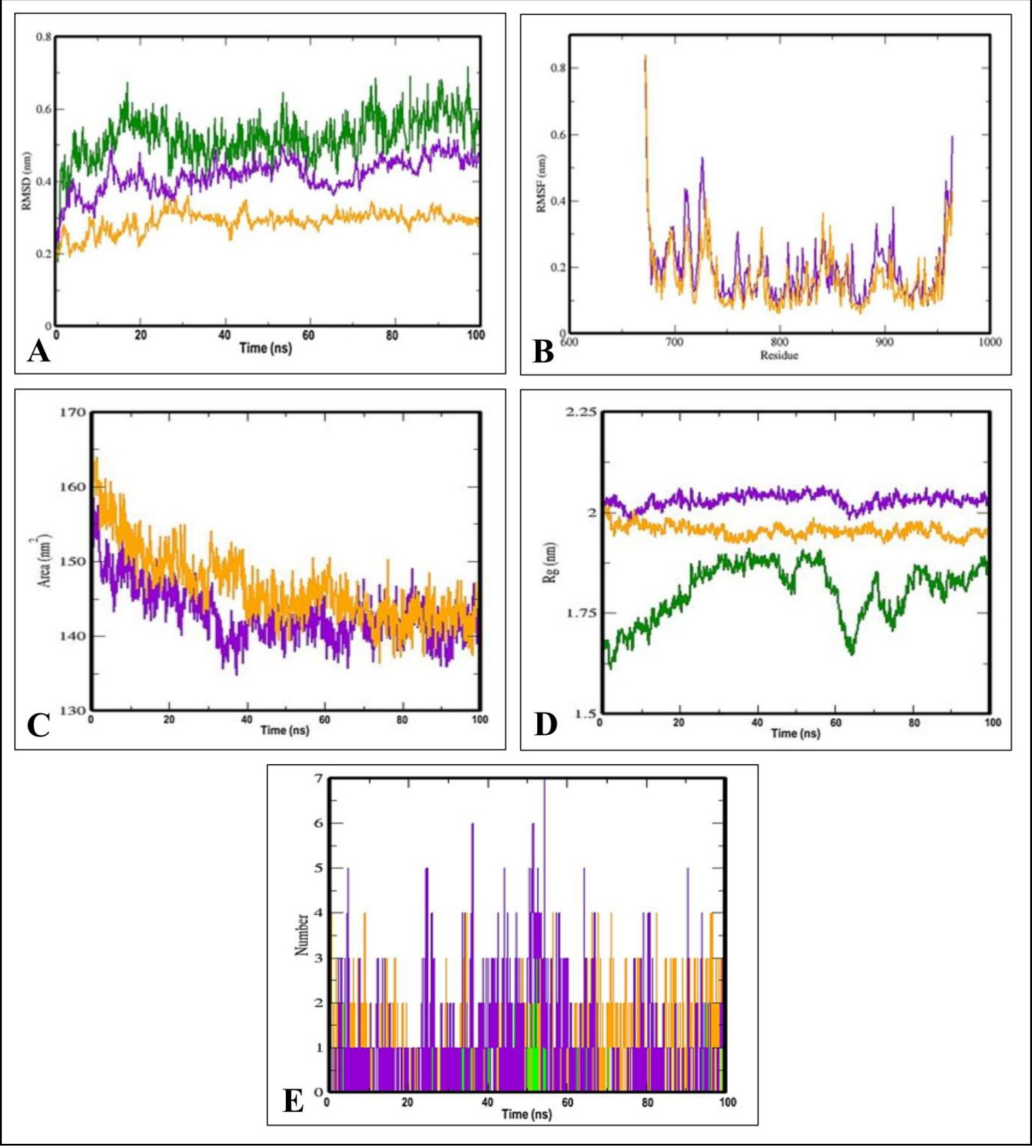
Visualization of MD simulation trajectory plots of EGFR tyrosine kinase domain in unbound, lead metabolite (Aspochalasin M) bound and standard inhibitor (Erlotinib) bound complex during 100 ns MD simulation period. A: RMSD; B: RMSF; C: SASA; D: Rg and E: Hydrogen bond formation. Green: unbound EGFR tyrosine kinase domain, orange: EGFR tyrosine kinase domain-Aspochalasin M complex and purple: EGFR tyrosine kinase domain-Erlotinib complex.

The RMSD is a common metric that used in MD simulations to assess the stability of a protein or protein-ligand complex over time [42]. A lower RMSD value indicates greater stability. The RMSD plot for the EGFR-Erlotinib complex showed that it became stable at around 40 ns into the simulation. This suggests that the EGFR-Erlotinib complex reached a relatively stable conformation at this point and remained stable thereafter. In contrast, the RMSD graphs for the EGFR-Aspochalasin M and EGFR-Erlotinib complexes showed a divergence from the protein plot’s orientation at around 30 ns. This divergence indicates that both complexes started to deviate from their initial conformations and became less stable. The EGFR-Aspochalasin M complex exhibited greater stability in the binding site compared to the EGFR-Erlotinib complex. This suggests that, despite the initial divergence, the EGFR-Aspochalasin M complex eventually reached a stable conformation that was more favorable in the binding site compared to the EGFR-Erlotinib complex (Fig 6A).

The RMSF is a measure used to assess the flexibility or fluctuation of individual atoms or residues within a protein structure during a simulation [43]. It helps identify regions of the protein that experience the most or least structural variation compared to the mean structure. The C-terminal region of the protein showed the larger fluctuations in RMSF which suggests that this part of the protein experienced significant structural variability during the simulation. The RMSF plots for the compounds (Aspochalasin M and Erlotinib) displayed minor oscillations throughout the simulation which means that the compounds remained relatively stable in terms of their interactions with the protein, with only small fluctuations in their positions within the binding site. When comparing the two complexes, the EGFR-Aspochalasin M complex exhibited less fluctuation (i.e., greater stability) compared to the EGFR-Erlotinib complex. This indicates that Aspochalasin M had a more stable binding within the pockets of EGFR, with fewer variations in its position during the simulation (Fig 6B).

The Rg is a measure that used in MD simulations to assess the compactness or spatial distribution of atoms within a molecule or a region of a protein [44]. It calculates the root mean square distance of all atoms from their common center of mass by taking into account their masses and positions. The Rg accounts for the various masses of atoms when calculating the distances and considers the folding, shape, and flexibility of the molecule or protein at each time step during the simulation trajectory. It also accounts for rotational motion around an axis. During the Rg analysis, it was observed that Aspochalasin M was tightly packed inside the binding pockets of EGFR. This suggests that Aspochalasin M maintained a compact and stable conformation within the binding site of the EGFR protein throughout the simulation (Fig 6C).

The SASA is a measure that used in MD simulations to calculate the exposed surface area of a molecule, often a protein or protein-ligand complex, to the surrounding solvent molecules [45]. The SASA analysis focuses on calculating the circumference or surface area of the hydrophobic cores within the protein-ligand complexes. Hydrophobic cores are typically regions of a protein or complex that contain hydrophobic (non-polar) amino acids and are involved in ligand binding. It is particularly useful for understanding the hydrophobic and hydrophilic regions of a molecule. The SASA for the protein-ligand complexes decreased which indicates that the protein and ligands (Aspochalasin M and Erlotinib) interacted effectively within the inhibitor’s binding site. This decrease in SASA suggests that the complexes became more compact or buried in the binding site due to their interaction. The EGFR bound-Aspochalasin M complex and EGFR bound-Erlotinib complex showed contemporaneous plots on the SASA plots. This suggests that both complexes exhibited similar behaviour in terms of SASA during the simulation. However, the plot further suggests that the EGFR bound-Aspochalasin M complex had a more effective capability for binding compared to the EGFR bound-Erlotinib complex (Fig 6D).

The hydrogen bonds are important interactions between atoms in molecules, and in the context of MD simulations, they can reveal the stability of ligand-protein interactions [31]. According to the analysis, the hydrogen bonds between the ligands and specific amino acid residues (THR766, ASP817, MET769) were consistently present throughout the entire duration of the simulation being investigated. This suggests that these interactions were stable and enduring over time. Aspochalasin M was found to form two hydrogen bonds, one with THR766 and another with ASP817. On the other hand, Erlotinib formed one hydrogen bond with MET769. It is important to note that the analysis focused on intermolecular hydrogen bonds between the ligands and the relevant amino acid residues which means that only hydrogen bonds directly involved in the ligand-protein interactions were considered. Based on the analysis, it appears that Aspochalasin M can potentially form a maximum of seven hydrogen bonds, while Erlotinib can form up to three hydrogen bonds. This suggests that Aspochalasin M has a greater capacity for hydrogen bond interactions with the protein compared to Erlotinib (Fig 6E). The MD simulation at 100 ns showed that Tupichinols E could stabilize the protein structure when it binds to EGFR tyrosine kinase [35].

### MM-PBSA analysis

Information regarding the degree of ligand affinity for the protein can be determined by analyzing the binding free energy. This energy is the difference in free energy between the completely ligand-bound and unbound states [46] (Dong et al., 2021). Further, the MM-PBSA analysis of the lead metabolite (Aspochalasin M)/ standard inhibitor (Erlotinib)–bound EGFR tyrosine kinase domain complex trajectories of the last 20 ns (80–100 ns) MD simulations were performed to determine the complex’s thermodynamics parameters such as van der Waals energy (E_vdw_), electrostatic energy (E_Elec_), polar solvation energy (ΔE_Polar_), SASA energy and binding free energy (ΔE_Binding_), all of which are expressed as kcal/mol (Table 3). Compared to other energies, van der Waals energy was the main driver of complex formation [47, 48] and it can be inferred that the binding energy and van der Waals energy significantly influenced the complex formation. Based on energy calculation, the expected outcomes were largely beneficial from an energetic standpoint. Additionally, it was discovered that the EGFR bound-Aspochalasin M complex had larger (more negative) binding free energies than the EGFR bound-Erlotinib complex, indicating that it is more stable and would need more energy to dissociate. Our findings corroborate the outcomes of molecular docking and MD simulations regarding the compound’s overall binding efficiency.

**Table 3:** Binding free energy calculations of EGFR tyrosine kinase complexed with the lead metabolite of *Trichoderma* spp. and standard inhibitor.

Category	1M17-Aspochalasin M complex	1M17-Erlotinib complex
	Values (kcal/mol)	Values (kcal/mol)
Van der Waal’s energy	– 103.746	– 88.198
Electrostatic energy	– 3.672	– 5.564
Polar salvation energy	26.164	20.810
SASA energy	– 8.120	– 5.245
Binding energy	– 79.440	– 47.338

## Conclusion

Small molecule natural products such as anEGFR tyrosine kinase inhibiting drug have attracted great interest for cancer treatment since these molecules could potentially have a high affinity for and inhibit the enzyme with minimal side effects. Therefore, the efficiency of secondary metabolites of *Trichoderma* spp. was explored for suppressing the EGFR tyrosine kinase to the current regime of cancer therapeutics in the present study through *in silico* molecular docking, MD simulation and MM-PBSA approach. Among 200 secondary metabolites, three leading metabolites (Harzianelactone A, Pretrichodermamide G and Aspochalasin M) demonstrated the least binding energy to EGFR tyrosine kinase than the standard EGFR inhibitor (Erlotinib). In addition, the MD simulation studies confirmed the stability of the EGFR tyrosine kinase–Aspochalasin M complex. The pharmacokinetic and toxicity properties further confirmed the efficiency of Aspochalasin M as a non-mutagenic with no harmful side effects (*viz*., hERG toxicity, hepatotoxicity and skin sensitization) and exhibited favourable logBB value. In addition, the BOILED-Egg model was predicted that Aspochalasin M has superior absorption in the digestive system and removed from the CNS. The positive bioavailability score (0.55) of Aspochalasin M might improve its absorption in the digestive tract when administered orally. In conclusion, Aspochalasin M could be an excellent drug-like candidate for cancer treatment. Furthermore, additional *in vitro* and *in vivo* animal studies are required to validate the anticancer effects of Aspochalasin M present in *Trichoderma* spp.

## Supporting information

S1 Table Docking score of secondary metabolites of *Trichoderma* spp. against EGFR tyrosine kinase domain. (DOCX)

## Abbreviations

ADMET: Absorption, distribution, metabolism, excretion and toxicity
CNS: Central nervous system
EGFR: Epidermal growth factor receptor
GROMACS: Groningen Machine for Chemical Simulations
INSOLU: Insolubility
INSATU: In saturation
kcal/mol: kilocalories per mole
LIPO: Lipophilicity
MD: Molecular dynamics
MM-PBSA: Molecular Mechanics Poisson−Boltzmann Surface Area
ps: Picoseconds
PDB: Protein Data Bank
OCT2: Organic cation transporter 2
RCSB PDB: Research Collaboratory for Structural Bioinformatics Protein Data Bank
RMSD: Root Mean Square Deviation
RMSF: Root Mean Square Fluctuation
Rg: Radius of Gyration
SASA: Solvent Accessible Surface Area
SDF: Structure Data File
VDss: Volume of distribution
2D: Two-dimensional
3D: Three-dimensional

## References

[1] Xu N, Fang W, Mu L, Tang Y, Gao L, Ren S, et al. Overexpression of wildtype EGFR is tumorigenic and denotes a therapeutic target in non-small cell lung cancer. Oncotarget. 2016; 7:3884–3896. doi: 10.18632/oncotarget.6461 26646697 PMC4826177

[2] Sigismund S, Avanzato D, Lanzetti L. Emerging functions of the EGFR in cancer. Molecular Oncology. 2018; 12:3–20. doi: 10.1002/1878-0261.12155 29124875 PMC5748484

[3] Vallböhmer D, Lenz H-J. Epidermal growth factor receptor as a target for chemotherapy. Clinical Colorectal Cancer. 2005; 5:S19–S27. 10.3816/CCC.2005.s.003 15871762

[4] Choowongkomon K, Sawatdichaikul O, Songtawee N, Limtrakul J. Receptor-based virtual screening of EGFR kinase inhibitors from the NCI diversity database. Molecules. 2010; 15:4041–4054. doi: 10.3390/molecules15064041 20657425 PMC6264413

[5] Uribe ML, Marrocco I, Yarden Y. EGFR in cancer: Signaling mechanisms, drugs, and acquired resistance. Cancers. 2021; 13:2748. doi: 10.3390/cancers13112748 34206026 PMC8197917

[6] Abourehab MAS, Alqahtani AM, Youssif BGM, Gouda AM. Globally approved EGFR inhibitors: Insights into their syntheses, target kinases, biological activities, receptor interactions, and metabolism. Molecules. 2021; 26:6677. doi: 10.3390/molecules26216677 34771085 PMC8587155

[7] Zubair T, Bandyopadhyay D. Small molecule EGFR inhibitors as anticancer agents: Discovery, mechanisms of action, and opportunities. International Journal of Molecular Sciences. 2023; 24:2651. doi: 10.3390/ijms24032651 36768973 PMC9916655

[8] Raymond E, Faivre S, Armand JP. Epidermal growth factor receptor tyrosine kinase as a target for anticancer therapy. Drugs. 2000; 60:15–23. doi: 10.2165/00003495-200060001-00002 11129168

[9] Kobayashi S, Boggon TJ, Dayaram T, Janne PA, Kocher O, Meyerson M, et al. EGFR mutation and resistance of non-small-cell lung cancer to gefitinib. New England Journal of Medicine. 2005; 352:786–792. doi: 10.1056/NEJMoa044238 15728811

[10] Pao W, Miller VA, Politi KA, Riely GJ, Somwar R, Zakowski MF, et al. Acquired resistance of lung adenocarcinomas to gefitinib or Erlotinib is associated with a second mutation in the EGFR kinase domain. PLOS Medicine. 2005; 2:e73. doi: 10.1371/journal.pmed.0020073 15737014 PMC549606

[11] Niveshika, Verma E, Maurya SK, Mishra R, Mishra AK. The combined use of in silico, in vitro, and in vivo analyses to assess anti-cancerous potential of a bioactive compound from Cyanobacterium Nostoc sp. MGL001. Frontiers in Pharmacology. 2017; 8:873. doi: 10.3389/fphar.2017.00873 29230175 PMC5711831

[12] Anandan S, Gowtham HG, Shivakumara CS, Thampy A, Brijesh Singh S, Murali M, et al. Integrated approach for studying bioactive compounds from *Cladosporium* spp. against estrogen receptor alpha as breast cancer drug target. Scientific Reports. 2022; 12:22446. 10.1038/s41598-022-22038-x 36575224 PMC9794773

[13] Singh BP, Rateb ME, Rodriguez-Couto S, Polizeli MdLTdM, Li W-J. Editorial: microbial secondary metabolites: Recent developments and technological challenges. Frontiers in Microbiology. 2019; 10:914. doi: 10.3389/fmicb.2019.00914 31105684 PMC6498875

[14] Zhang JL, Tang WL, Huang QR, Li YZ, Wei ML, Jiang LL, et al. *Trichoderma*: A treasure house of structurally diverse secondary metabolites with medicinal importance. Frontiers in Microbiology. 2021; 12:723828. 10.3389/fmicb.2021.723828 34367122 PMC8342961

[15] Gowtham HG, Murali M, Singh SB, Shivamallu C, Pradeep S, Shivakumar CS, et al. Phytoconstituents of *Withania somnifera* unveiled Ashwagandhanolide as a potential drug targeting breast cancer: Investigations through computational, molecular docking and conceptual DFT studies. PLoS One. 2022; 17:e0275432. 10.1371/journal.pone.0275432 PMC9536605 36201520

[16] Murali M, Gowtham HG, Ansari MA, Alomary MN, Alghamdi S, Almehmadi M, et al. Repositioning therapeutics for SARS-CoV-2: Virtual screening of plant-based anti-HIV compounds as possible inhibitors against COVID-19 viral RdRp. Current Pharmaceutical Design. 2022a; 28:969–980. doi: 10.2174/1381612828666220428120939 35796443

[17] Murali M, Gowtham HG, Shilpa N, Krishnappa HKN, Ledesma AE, Jain AS, et al. Exploration of anti-HIV phytocompounds against SARS-CoV-2 main protease: Structure-based screening, molecular simulation, ADME analysis and conceptual DFT studies. Molecules. 2022b; 27:8288. doi: 10.3390/molecules27238288 36500380 PMC9736867

[18] O’Boyle NM, Banck M, James CA, Morley C, Vandermeersch T, Hutchison GR. Open Babel: An open chemical toolbox. Journal of Cheminformatics. 2011; 3:1–14. 10.1186/1758-2946-3-33 21982300 PMC3198950

[19] Stamos J, Sliwkowski MX, Eigenbrot C. Structure of the epidermal growth factor receptor kinase domain alone and in complex with a 4-anilinoquinazoline inhibitor. Journal of Biological Chemistry.2002; 277:46265–46272. doi: 10.1074/jbc.M207135200 12196540

[20] Pettersen EF, Goddard TD, Huang CC, Couch GS, Greenblatt DM, Meng EC, et al. UCSF Chimera—A visualization system for exploratory research and analysis. Journal of Computational Chemistry.2004; 25:1605–1612. doi: 10.1002/jcc.20084 15264254

[21] Eberhardt J, Santos-Martins D, Tillack AF, Forli S. AutoDock Vina 1.2.0: New docking methods, expanded force field, and python bindings. Journal of Chemical Information and Modeling. 2021; 61:3891–3898. doi: 10.1021/acs.jcim.1c00203 34278794 PMC10683950

[22] Pires DEV, Blundell TL, Ascher DB. pkCSM: Predicting small-molecule pharmacokinetic and toxicity properties using graph-based signatures. Journal of Medicinal Chemistry. 2015; 58:4066–4072. doi: 10.1021/acs.jmedchem.5b00104 25860834 PMC4434528

[23] Daina A, Michielin O, Zoete V. SwissADME: A free web tool to evaluate pharmacokinetics, drug-likeness and medicinal chemistry friendliness of small molecules. Scientific Reports.2017; 7:42717. doi: 10.1038/srep42717 28256516 PMC5335600

[24] Dharmashekara C, Pradeep S, Prasad SK, Jain AS, Syed A, Prasad KS, et al. Virtual screening of potential phyto-candidates as therapeutic leads against SARS-CoV-2 infection. Environmental Challenges. 2021; 4:100136. 10.1016/j.envc.2021.100136 PMC8110638 38620722

[25] Prasad SK, Pradeep S, Shimavallu C, Kollur SP, Syed A, Marraiki N, et al. Evaluation of *Annona muricata* Acetogenins as potential anti-SARS-CoV-2 agents through computational approaches. Frontiers in Chemistry. 2021; 8:624716. 10.3389/fchem.2020.624716 33732682 PMC7958878

[26] Pradeep S, Patil SM, Dharmashekara C, Jain A, Ramu R, Shirahatti PS, et al. Molecular insights into the *in silico* discovery of corilagin from *Terminalia chebula* as a potential dual inhibitor of SARS-CoV-2 structural proteins. Journal of Biomolecular Structure and Dynamics. 2022; 2022:1–16. 10.1080/07391102.2022.2158943 36576118

[27] Chadha N, Tiwari AK, Kumar V, Milton MD, Mishra AK. *In silico* thermodynamics stability change analysis involved in BH4 responsive mutations in phenylalanine hydroxylase: QM/MM and MD simulations analysis. Journal of Biomolecular Structure and Dynamics. 2015; 33:573–583. 10.1080/07391102.2014.897258 24628256

[28] Valdés-Tresanco MS, Valdés-Tresanco ME, Valiente PA, Moreno E. gmx_MMPBSA: A new tool to perform end-state free energy calculations with GROMACS. Journal of Chemical Theory and Computation. 2021; 17:6281–6291. doi: 10.1021/acs.jctc.1c00645 34586825

[29] Gowtham HG, Ahmed F, Anandan S, Shivakumara CS, Bilagi A, Pradeep S, et al. In silico computational studies of bioactive secondary metabolites from Wedelia trilobata against anti-apoptotic B-cell lymphoma-2 (Bcl-2) protein associated with cancer cell survival and resistance. Molecules. 2023; 28:1588. doi: 10.3390/molecules28041588 36838574 PMC9959492

[30] Hospital A, Goñi JR, Orozco M, Gelpi J. Molecular dynamics simulations: advances and applications. Advances and Applications in Bioinformatics and Chemistry. 2015; 8:37–47. doi: 10.2147/AABC.S70333 26604800 PMC4655909

[31] Coimbra JTS, Feghali R, Ribeiro RP, Ramos MJ, Fernandes PA. The importance of intramolecular hydrogen bonds on the translocation of the small drug Piracetam through a lipid bilayer. RSC Advances. 2021; 11:899–908. doi: 10.1039/D0RA09995C 35423709 PMC8693363

[32] Patil R, Das S, Stanley A, Yadav L, Sudhakar A, Varma AK. Optimized hydrophobic interactions and hydrogen bonding at the target-ligand interface leads the pathways of drug-designing. PLoS One. 2010; 5:e12029. doi: 10.1371/journal.pone.0012029 20808434 PMC2922327

[33] Ramírez D, Caballero J. Is it reliable to take the molecular docking top scoring position as the best solution without considering available structural data? Molecules. 2018; 23:1038. doi: 10.3390/molecules23051038 29710787 PMC6102569

[34] Shah A, Seth AK. *In silico* identification of novel flavonoids targeting epidermal growth factor receptor. Current Drug Discovery Technologies. 2021; 18:75–82. 10.2174/1570163816666191023102112 31657688

[35] Sepay N, Mondal R, Al-Muhanna MK, Saha D. Identification of natural flavonoids as novel EGFR inhibitors using DFT, molecular docking, and molecular dynamics. New Journal of Chemistry. 2022; 46:9735–9744. 10.1039/D2NJ00389A

[36] Suriya U, Mahalapbutr P, Wimonsong W, Yotphan S, Choowongkomon K, Rungrotmongkol T. Quinoxalinones as a novel inhibitor scaffold for EGFR (L858R/T790M/C797S) tyrosine kinase: Molecular docking, biological evaluations, and computational insights. Molecules. 2022; 27:8901. doi: 10.3390/molecules27248901 36558033 PMC9788584

[37] Boström J, Hogner A, Schmitt S. Do structurally similar ligands bind in a similar fashion? Journal of Medicinal Chemistry. 2006; 49:6716–6725. doi: 10.1021/jm060167o 17154502

[38] Ehmki ESR, Rarey M. Exploring structure-activity relationships with three-dimensional matched molecular pairs-A review. ChemMedChem. 2018; 13:482–489. doi: 10.1002/cmdc.201700628 29211343

[39] Pantaleão SQ, Fernandes PO, Gonçalves JE, Maltarollo VG, Honorio KM. Recent advances in the prediction of pharmacokinetics properties in drug design studies: A review. ChemMedChem. 2022; 17:e202100542. doi: 10.1002/cmdc.202100542 34655454

[40] Poulin P. Drug distribution to human tissues: Prediction and examination of the basic assumption in in vivo pharmacokinetics-pharmacodynamics (PK/PD) Research. Journal of Pharmaceutical Sciences. 2015; 104:2110–2118. doi: 10.1002/jps.24427 25808270

[41] Aungst BJ. Optimizing oral bioavailability in drug discovery: An overview of design and testing strategies and formulation options. Journal of Pharmaceutical Sciences. 2017; 106:921–929, doi: 10.1016/j.xphs.2016.12.002 27986598

[42] Sargsyan K, Grauffel C, Lim C. How molecular size impacts RMSD applications in molecular dynamics simulations. Journal of Chemical Theory and Computation. 2017; 13:1518–1524. doi: 10.1021/acs.jctc.7b00028 28267328

[43] Boroujeni MB, Dastjerdeh MS, Shokrgozar MA, Rahimi H, Omidinia E. Computational driven molecular dynamics simulation of keratinocyte growth factor behavior at different pH conditions. Informatics in Medicine Unlocked. 2021; 23:100514. 10.1016/j.imu.2021.100514

[44] Lobanov MY, Bogatyreva NS, Galzitskaya OV. Radius of gyration as an indicator of protein structure compactness. Molecular Biology. 2008; 42:623–628. 10.1134/s0026893308040195 18856071

[45] Ali SA, Hassan MI, Islam A, Ahmad F. A review of methods available to estimate solvent-accessible surface areas of soluble proteins in the folded and unfolded states. Current Protein & Peptide Science. 2014; 15:456–476. 10.2174/1389203715666140327114232 24678666

[46] Dong L, Qu X, Zhao Y, Wang B. Prediction of binding free energy of protein-ligand complexes with a hybrid Molecular Mechanics/Generalized Born Surface Area and Machine Learning Method. ACS Omega. 2021; 6:32938– 32947. doi: 10.1021/acsomega.1c04996 34901645 PMC8655939

[47] Dasmahapatra U, Kumar CK, Das S, Subramanian PT, Murali P, Isaac AE, et al. In-silico molecular modelling, MM/GBSA binding free energy and molecular dynamics simulation study of novel pyrido fused imidazo[4,5-c]quinolines as potential anti-tumor agents. Frontiers in Chemistry. 2022; 10:991369. doi: 10.3389/fchem.2022.991369 36247684 PMC9566731

[48] Murali M, Ahmed F, Gowtham HG, Aribisala JO, Abdulsalam RA, Shati AA, et al. Exploration of CviR-mediated quorum sensing inhibitors from Cladosporium spp. against Chromobacterium violaceum through computational studies. Scientific Reports. 2023; 13:15505. doi: 10.1038/s41598-023-42833-4 37726386 PMC10509224

