# Supplementary material for: Secondary metabolites of Trichoderma spp. as EGFR tyrosine kinase inhibitors: Evaluation of anticancer efficacy through computational approach
Source: PLoS One. 2024 Jan 24;19(1):e0296010. doi: 10.1371/journal.pone.0296010 (PMC10824427; doi:10.1371/journal.pone.0296010)
Supplement: S1 Table — (DOCX) [file pone.0296010.s001.docx]

**Supplementary Table 1. Docking score of secondary metabolites of *Trichoderma* spp. against EGFR tyrosine kinase domain.**

| **Sl. No.** | **Compounds*** | **PubChem ID/**  **ChemSpider ID** | **Docking score (kcal/mol)** |
| --- | --- | --- | --- |
| 1. | Trichodermarin G | ChemSpider ID 103704580 | – 6.2 |
| 2. | Trichodermarin H | ChemSpider ID 103704581 | – 6.3 |
| 3. | Trichodermarin I | ChemSpider ID 103704582 | – 6.5 |
| 4. | Trichodermarin J | ChemSpider ID 103704583 | – 6.1 |
| 5. | Trichodermarin K | ChemSpider ID 103704584 | – 6.6 |
| 6. | Trichodermarin L | ChemSpider ID 103704585 | – 7.2 |
| 7. | Trichodermarin M | ChemSpider ID 103704586 | – 7.5 |
| 8. | Trichodermarin N | ChemSpider ID 103704587 | – 6.9 |
| 9. | Trichodermol | CID_12315016 | – 6.4 |
| 10. | Trichodermin | CID_20806 | – 6.1 |
| 11. | *Trichoderminol | - | – 6.5 |
| 12. | Trichodermarin A | CID_145720818 | – 6.5 |
| 13. | Trichodermarin B | CID_145720819 | – 6.4 |
| 14. | *2,4,12-Trihydroxyapotrichothecene | - | – 7.2 |
| 15. | Trichobreol A | CID_156580685 | – 6.9 |
| 16. | Trichobreol B | CID_156580686 | – 6.8 |
| 17. | Trichobreol C | CID_156580682 | – 6.9 |
| 18. | Trichobreol D | ChemSpider ID 95798020 | – 7.4 |
| 19. | Trichobreol E | ChemSpider ID 95798021 | – 6.2 |
| 20. | Harzianum A | CID_10453605 | – 7.5 |
| 21. | Harzianum B | CID_6444304 | – 8.1 |
| 22. | Trichothecinol A | CID_10472929 | – 6.8 |
| 23. | 8-Deoxytrichothecin | CID_70690346 | – 6.7 |
| 24. | Trichothecinol B | CID_10246280 | – 6.8 |
| 25. | Trichocarotin A | CID_156581630 | – 7.0 |
| 26. | Trichocarotin B | CID_156581631 | – 6.9 |
| 27. | Trichocarotin C | CID_147992277 | – 6.8 |
| 28. | Trichocarotin D | CID_156581632 | – 7.1 |
| 29. | Trichocarotin E | CID_148772264 | – 5.9 |
| 30. | Trichocarotin F | CID_156581633 | – 6.6 |
| 31. | Trichocarotin G | CID_156581634 | – 6.6 |
| 32. | Trichocarotin H | CID_148214470 | – 6.3 |
| 33. | CAF-603 | CID_147090 | – 7.1 |
| 34. | Trichocarane B | CID_10682391 | – 6.3 |
| 35. | 7-Beta-hydroxy CAF-603 | CID_72713582 | – 6.0 |
| 36. | Trichocarane A | CID_21606643 | – 6.6 |
| 37. | Trichocadinin A | CID_147140349 | – 7.1 |
| 38. | Trichodermaloid A | CID_156582486 | – 7.9 |
| 39. | Trichodermaloid B | CID_156582487 | – 7.5 |
| 40. | Trichodermaloid C | CID_156582488 | – 7.7 |
| 41. | Aspergilloid G | ChemSpider ID 90606660 | – 7.4 |
| 42. | Rhinomilisin E | CID_145720943 | – 6.5 |
| 43. | Rhinomilisin G | CID_145720945 | – 6.8 |
| 44. | 3,7,11-Trihydroxy-cycloneran | ChemSpider ID 90671133 | – 6.3 |
| 45. | (10*E*)-Isocyclonerotriol | CID_156582379 | – 6.8 |
| 46. | (10*Z*)-Isocyclonerotriol | CID_156582380 | – 6.7 |
| 47. | 11-Methoxy-9-cycloneren-3,7-diol | CID_156581678 | – 6.2 |
| 48. | 9-Cycloneren-3,7,11-triol | CID_139590988 | – 6.5 |
| 49. | *(–)-Cyclonerodiol | - | – 6.4 |
| 50. | Methyl 3,7-dihydroxy-15-cycloneranate | CID_156581680 | – 6.2 |
| 51. | 10-Cycloneren-3,5,7-triol | CID_156581679 | – 6.4 |
| 52. | *10(*E*)-Cyclonerotriol | - | – 6.4 |
| 53. | Neomacrophorin I | CID_74538726 | – 7.7 |
| 54. | Neomacrophorin II | CID_102236951 | – 7.4 |
| 55. | Neomacrophorin III | CID_102236952 | – 7.8 |
| 56. | 3-Deoxyneomacrophorin IV | CID_146682215 | – 7.2 |
| 57. | 3-Oxoneomacrophorin I | CID_146682216 | – 8.3 |
| 58. | 3-Oxoneomacrophorin II | CID_146682217 | – 8.5 |
| 59. | Neomacrophorin VII | CID_146682218 | – 8.4 |
| 60. | 5’-Epimacrophorin B | CID_146682213 | – 8.1 |
| 61. | 5’-Deoxyneomacrophorin IV | CID_146682219 | – 7.5 |
| 62. | Premacrophorin III | CID_146682220 | – 7.3 |
| 63. | Premacrophorindiol | CID_146682221 | – 7.6 |
| 64. | Premacrophorintriol I | CID_146682222 | – 7.3 |
| 65. | Premacrophorintriol II | CID_146682214 | – 7.3 |
| 66. | Microsphaeropsisin B | CID_139590423 | – 6.6 |
| 67. | Microsphaeropsisin C | CID_139590424 | – 7.1 |
| 68. | 8-Acoren-3,11-diol | CID_156581681 | – 6.9 |
| 69. | Trichoacorenol | CID_10331156 | – 6.2 |
| 70. | Trichoacorenol B | CID_139591328 | – 6.2 |
| 71. | Trichodermene A | ChemSpider ID 81424708 | – 7.0 |
| 72. | Trichoderiol A | ChemSpider ID 28289058 | – 6.6 |
| 73. | *Trichoderiol B | - | – 6.8 |
| 74. | Trichocuparin A | ChemSpider ID 103704588 | – 7.3 |
| 75. | Trichocuparin B | ChemSpider ID 103704589 | – 7.0 |
| 76. | Trichodone A | CID_139586090 | – 6.4 |
| 77. | Trichodone B | CID_139587949 | – 6.7 |
| 78. | *Trichodone C | - | – 6.5 |
| 79. | *Atrichodermone C | - | – 6.3 |
| 80. | Harzianoic acid A | CID_146682324 | – 6.2 |
| 81. | Harzianoic acid B | CID_146682325 | – 6.5 |
| 82. | Harzianol F | CID_156582616 | – 6.7 |
| 83. | Harzianol G | CID_156582617 | – 6.7 |
| 84. | Harzianol H | CID_156582618 | – 6.8 |
| 85. | Harzianol I | CID_154720051 | – 6.7 |
| 86. | Harzianol J | CID_156582619 | – 7.2 |
| 87. | 3*S*-Hydroxyharzianone | CID_146682504 | – 7.1 |
| 88. | Harziandione | CID_60203966 | – 8.0 |
| 89. | *Harzianol A | - | – 7.6 |
| 90. | Deoxytrichodermaerin | ChemSpider ID 103708816 | – 8.0 |
| 91. | Harzianone E | ChemSpider ID 90671132 | – 7.4 |
| 92. | 3*R*-Hydroxy-9*R*,10*R*-dihydroharzianone | CID_156581676 | – 7.0 |
| 93. | (9*R*,10*R*)-Dihydro-harzianone | CID_155526718 | – 7.8 |
| 94. | Harzianelactone | CID_156581284 | – 7.5 |
| 95. | Harzianelactone A | CID_156582052 | – 9.0 |
| 96. | Harzianelactone B | CID_156582053 | – 8.6 |
| 97. | Harzianone A | CID_156582054 | – 7.2 |
| 98. | Harzianone B | CID_156582055 | – 8.2 |
| 99. | Harzianone C | CID_156582056 | – 7.8 |
| 100. | Harzianone D | CID_156582057 | – 7.8 |
| 101. | Harziane | CID_156582058 | – 7.2 |
| 102. | Trichodermaerin | CID_139086489 | – 6.9 |
| 103. | Citrinovirin | CID_156581283 | – 7.2 |
| 104. | 11*R*-Methoxy-5,9,13-proharzitrien-3-ol | CID_156581677 | – 6.9 |
| 105. | Harzianolic acid A | ChemSpider ID 90671131 | – 7.1 |
| 106. | Trichodestruxin A | CID_156580993 | – 7.4 |
| 107. | Trichodestruxin B | CID_156580994 | – 6.9 |
| 108. | Trichodestruxin C | CID_156580995 | – 7.6 |
| 109. | Trichodestruxin D | CID_156580996 | – 7.2 |
| 110. | Destruxin E2 chlorohydrin | CID_132495843 | – 7.4 |
| 111. | *Destruxin A2 | - | – 7.9 |
| 112. | Homodestcardin | CID_139586683 | – 7.0 |
| 113. | Trichomide B | CID_102435899 | – 7.2 |
| 114. | Homodestruxin B | CID_163819 | – 7.1 |
| 115. | PF1022F | CID_101062244 | – 7.5 |
| 116. | Halobacillin | CID_139584620 | – 6.6 |
| 117. | Pretrichodermamide A | CID_129713921 | – 7.2 |
| 118. | Gliovirin | CID_85148368 | – 7.4 |
| 119. | Trichodermamide A | CID_10982906 | – 8.8 |
| 120. | *DC1149B | - | – 8.5 |
| 121. | Trichodermamide B | CID_639644 | – 8.6 |
| 122. | *DC1149R | - | – 8.4 |
| 123. | Iododithiobrevamide | CID_139587030 | – 7.6 |
| 124. | Chlorotrithiobrevamide | CID_139585992 | – 7.7 |
| 125. | Dithioaspergillazine A | CID_132496587 | – 8.1 |
| 126. | 5-*Epi*-pretrichodermamide A | CID_156580683 | – 7.5 |
| 127. | 5-*Epi*-trithiopretrichodermamide A | CID_156580684 | – 7.4 |
| 128. | *Pretrichodermamide G |  | – 9.1 |
| 129. | Trichodermamide G | ChemSpider ID 92172146 | – 7.9 |
| 130. | *Aspergillazin A | - | – 7.7 |
| 131. | Dehydroxymethylbis(dethio)bis(methylthio)gliotoxin | CID_146683985 | – 6.1 |
| 132. | (3S,6R)-6-(para-hydroxybenzyl)-1,4-dimethyl-3,6-bis(methylthio)piperazine-2,5-dione | CID_146683984 | – 6.0 |
| 133. | *Cyclo(L-5-MeO-Pro-L-5-MeO-Pro) | - | – 6.3 |
| 134. | *Trichothioneic acid | - | – 8.2 |
| 135. | Ethyl 2-bromo-4-chloroquinoline-3-carboxylate | CID_54486725 | – 6.6 |
| 136. | *Trichoderamide A | - | – 6.7 |
| 137. | *Trichoderamide B | - | – 6.8 |
| 138. | Trichodin A | CID_139584390 | – 8.9 |
| 139. | Harzianic acid | CID_54691490 | – 6.7 |
| 140. | *Atrichodermone A | - | – 6.4 |
| 141. | *5’-Acetoxy-deoxycyclonerin B | - | – 6.8 |
| 142. | *5’-Acetoxy-deoxycyclonerin D | - | – 7.2 |
| 143. | *Trichoharzin B | - | – 7.2 |
| 144. | *Methyl-trichoharzin | - | – 7.2 |
| 145. | Trichoharzin | CID_76853359 | – 7.2 |
| 146. | Eujavanicol A | CID_23643941 | – 6.5 |
| 147. | Trichoharzianol | CID_139584685 | – 6.8 |
| 148. | Trichodermic acid A | CID_101583709 | – 7.8 |
| 149. | Trichodermic acid B | CID_101583710 | – 7.1 |
| 150. | **Ent*-koninginin A | - | – 6.0 |
| 151. | *1,6-Di-epi-koninginin A | - | – 6.9 |
| 152. | *15-Hydroxykoninginin A | - | – 7.0 |
| 153. | *10-Deacetylkoningiopisin D | - | – 6.1 |
| 154. | Koninginin T | CID_156582571 | – 6.8 |
| 155. | *Koninginin L | - | – 6.4 |
| 156. | Trichoketide A | CID_122372540 | – 6.5 |
| 157. | Koninginin I | CID_102236896 | – 7.3 |
| 158. | Koninginin J | CID_102236897 | – 6.6 |
| 159. | *Koninginin K | - | – 6.4 |
| 160. | 7-O-methylkoninginin D | CID_46833400 | – 6.8 |
| 161. | Trichodermaketone A | CID_46833401 | – 6.3 |
| 162. | Trichodermaketone B | CID_46833709 | – 6.7 |
| 163. | Trichodermaketone C | CID_46833711 | – 6.8 |
| 164. | Trichodermaketone D | CID_46833710 | – 6.6 |
| 165. | Trichoketide A | CID_122372540 | – 6.7 |
| 166. | Trichoketide B | CID_122372541 | – 6.3 |
| 167. | *Koninginin M | - | – 6.4 |
| 168. | Trichoderone A | CID_57413333 | – 7.8 |
| 169. | Trichoderone B | CID_57413334 | – 7.9 |
| 170. | Aspochalasin D | CID_20839478 | – 8.0 |
| 171. | Aspochalasin J | CID_21576439 | – 8.2 |
| 172. | Aspochalasin I | CID_21576438 | – 7.7 |
| 173. | *Trichalasin C | - | – 8.1 |
| 174. | *Trichalasin D | - | – 7.5 |
| 175. | Aspochalasin M | CID_137700389 | – 9.4 |
| 176. | Aspochalasin P | CID_44471449 | – 7.9 |
| 177. | *Atrichodermone B | - | – 4.7 |
| 178. | 5-Hydroxycyclopenicillone | CID_139590516 | – 5.9 |
| 179. | *Xylogibloactone A | - | – 5.6 |
| 180. | *Xylogibloactone B | - | – 5.8 |
| 181. | Trichoderpyrone | CID_139589739 | – 6.4 |
| 182. | Trichoderone | CID_44820524 | – 4.9 |
| 183. | Trichophenol A | CID_156582381 | – 8.7 |
| 184. | 5-Hydroxy-3-hydroxymethyl-2-methyl-7-methoxychromone | CID_15434232 | – 6.9 |
| 185. | 4,6-Dihydroxy-5-methylphthalide | CID_14824644 | – 6.1 |
| 186. | * (*R*,3*E*,5*E*)-1-(3,5-dihydroxy-2,4-dimethylphenyl)-1-hydroxyhepta-3,5-dien-2-one | - | – 6.9 |
| 187. | * (*R*,3*E*,5*E*)-1-(3,5-dihydroxy-2,4-dimethylphenyl)-1-methoxyhepta-3,5-dien-2-one | - | – 6.6 |
| 188. | *Azaphilone D | - | – 6.6 |
| 189. | *Azaphilone E | - | – 7.2 |
| 190. | Cremenolide | CID_139586226 | – 7.2 |
| 191. | (3*R*,7*R*)-7-hydroxy-de-*O*-methyllasiodiplodin | CID_139590425 | – 8.4 |
| 192. | (3*R*)-5-oxo-de-*O*-methyllasiodiplodin | CID_139590426 | – 8.5 |
| 193. | Nafuredin C | ChemSpider ID 92172145 | – 7.4 |
| 194. | *Nafuredin A | - | – 7.4 |
| 195. | *Thioporidiol A | - | – 6.6 |
| 196. | *Thioporidiol B | - | – 6.5 |
| 197. | Violaceol I | CID_100615 | – 7.2 |
| 198. | Violaceol II | CID_16196968 | – 6.9 |
| 199. | *Trichodenol A | - | – 6.4 |
| 200. | Trichodenol B | CID_132556991 | – 6.7 |
| 201. | Erlotinib [Positive control] | CID_176870 | – 7.3 |

*****Chemical structuresof secondary metabolites were not found in any of the databases and were drawn using MarvinSketch software, according to Zhang et al. (2021) and used throughout the studies.
